# Human genetics influences microbiome composition involved in asthma exacerbations despite inhaled corticosteroid treatment

**DOI:** 10.1016/j.jaci.2023.05.021

**Published:** 2023-06-08

**Authors:** Javier Perez-Garcia, Antonio Espuela-Ortiz, José M. Hernández-Pérez, Ruperto González-Pérez, Paloma Poza-Guedes, Elena Martin-Gonzalez, Celeste Eng, Olaia Sardón-Prado, Elena Mederos-Luis, Paula Corcuera-Elosegui, Inmaculada Sánchez-Machín, Javier Korta-Murua, Jesús Villar, Esteban G. Burchard, Fabian Lorenzo-Diaz, Maria Pino-Yanes

**Affiliations:** a Genomics and Health Group, Department of Biochemistry, Microbiology, Cell Biology and Genetics, Universidad de La Laguna (ULL); b Pulmonary Medicine Service, Hospital Universitario N.S de Candelaria, La Laguna, Tenerife; c Pulmonary Medicine Section, Hospital Universitario de La Palma, La Palma; d Severe Asthma Unit, Allergy Department, Hospital Universitario de Canarias, La Laguna, Tenerife; e Department of Medicine, University of California San Francisco (UCSF), San Francisco; f Division of Pediatric Respiratory Medicine, Hospital Universitario Donostia, San Sebastián; g Department of Pediatrics, University of the Basque Country (UPV/EHU), San Sebastián; h Allergy Department, Hospital Universitario de Canarias, La Laguna, Tenerife; i CIBER de Enfermedades Respiratorias, Instituto de Salud Carlos III, Madrid; j Multidisciplinary Organ Dysfunction Evaluation Research Network, Research Unit, Hospital Universitario Dr. Negrín, Las Palmas de Gran Canaria; k Li Ka Shing Knowledge Institute at the St. Michael’s Hospital, Toronto, Ontario; l Department of Bioengineering and Therapeutic Sciences, University of California San Francisco (UCSF), San Francisco; m Instituto Universitario de Enfermedades Tropicales y Salud Pública de Canarias, Universidad de La Laguna (ULL); n Instituto de Tecnologías Biomédicas, Universidad de La Laguna (ULL), La Laguna, Tenerife

**Keywords:** Airway microbiome, CEBP, gastroesophageal reflux disease, inhaled corticosteroids, mGWAS, NR3C1, NF-κB, obesity, smoking, trichostatin A

## Abstract

**Background:**

The upper-airway microbiome is involved in asthma exacerbations despite inhaled corticosteroid (ICS) treatment. Although human genetics regulates microbiome composition, its influence on asthma-related airway bacteria remains unknown.

**Objective:**

We sought to identify genes and biological pathways regulating airway-microbiome traits involved in asthma exacerbations and ICS response.

**Methods:**

Saliva, nasal, and pharyngeal samples from 257 European patients with asthma were analyzed. The association of 6,296,951 genetic variants with exacerbation-related microbiome traits despite ICS treatment was tested through microbiome genome-wide association studies. Variants with 1 × 10^−4^ < *P* < 1 × 10^−6^ were examined in gene-set enrichment analyses. Significant results were sought for replication in 114 African American and 158 Latino children with and without asthma. ICS-response–associated single nucleotide polymorphisms reported in the literature were evaluated as microbiome quantitative trait loci. Multiple comparisons were adjusted by the false discovery rate.

**Results:**

Genes associated with exacerbation-related airway-microbiome traits were enriched in asthma comorbidities development (ie, reflux esophagitis, obesity, and smoking), and were likely regulated by trichostatin A and the nuclear factor-κB, the glucocorticosteroid receptor, and CCAAT/enhancer-binding protein transcription factors (7.8 × 10^−13^ ≤ false discovery rate ≤ 0.022). Enrichment in smoking, trichostatin A, nuclear factor-κB, and glucocorticosteroid receptor were replicated in the saliva samples from diverse populations (4.42 × 10^−9^ ≤ *P* ≤ .008). The ICS-response–associated single nucleotide polymorphisms rs5995653 (*APOBEC3B-APOBEC3C*), rs6467778 (*TRIM24*), and rs5752429 (*TPST2*) were identified as microbiome quantitative trait loci of *Streptococcus*, *Tannerella*, and *Campylobacter* in the upper airway (0.027 ≤ false discovery rate ≤ 0.050).

**Conclusions:**

Genes associated with asthma exacerbation–related microbiome traits might influence asthma comorbidities. We reinforced the therapeutic interest of trichostatin A, nuclear factor-κB, the glucocorticosteroid receptor, and CCAAT/enhancer-binding protein in asthma exacerbations.

## Graphical Abstract

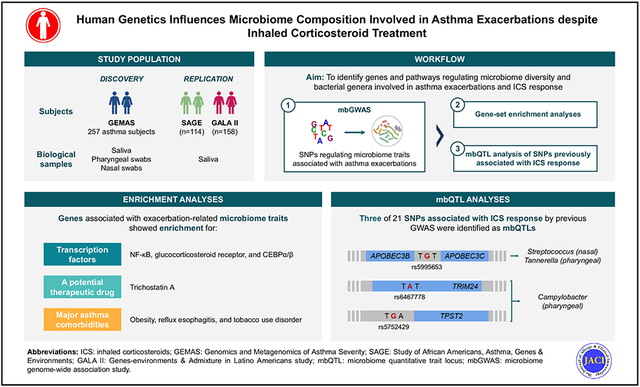

## INTRODUCTION

The human microbiome involves all the microorganisms inhabiting the human body.^1^ The elevated number of bacterial species and their genetic diversity at different body sites have aroused interest in investigating the influence of the microbiome on human diseases, especially in asthma and allergies.^1^ Previous studies reported that the airway and gut microbiome composition and microbial exposures during the lifespan contribute to asthma pathogenesis and treatment response.^1,2^ These microbial communities are influenced by host genetics and environmental factors, leading to high interindividual variation.^3^ However, little is known about host genetic variants modifying the composition of the upper-airway microbiome. Previous studies have reported both an important contribution of human genetics in the oral microbiome composition, but little effect on the nasal microbiome, and so its influence is not completely understood.^3-6^

Microbiome genome-wide association studies (mbGWAS) have revealed single nucleotide polymorphisms (SNPs) regulating the microbiome composition, known as microbiome quantitative trait loci (mbQTLs). However, most mbGWAS have focused on analyzing the gut microbiome, and other asthma-relevant tissues have been barely examined.^4,7^ To date, only 4 mbGWAS have investigated the influence of host genetics on the upper-airway microbiome.^3,5,6,8^ These studies have reported that mucosal immunity genes and immunity-related pathways are relevant to regulating the nasal microbiome.^3,8^ In addition, the heritability of the oral microbiome has been estimated at more than 50%, and the influence of host genetics on the oral microbiome is potentially higher than that of environmental factors.^5,6^

Nonetheless, there is no study evaluating the effect of genetic loci on airway bacteria previously related to asthma pathogenesis.^1^ Recently, our group has identified multiple upper-airway microbiome biomarkers with a protective role for asthma exacerbations despite inhaled corticosteroid (ICS) treatment.^2^ We hypothesize that host genetics regulate the upper-airway microbiome diversity and composition involved in asthma exacerbations and ICS response. This study aimed to identify genetic variants and biological pathways regulating airway microbiome traits involved in asthma exacerbations despite ICS treatment.

## RESULTS AND DISCUSSION

A schematic overview of the available data and workflow of this study is represented in Fig E1 (in the Online Repository available at www.jacionline.org). A total of 257 saliva, 232 pharyngeal, and 229 nasal samples from European individuals with asthma from the Genomics and Metagenomics of Asthma Severity (GEMAS) study were analyzed in the discovery phase.^9^ Their main demographic and clinical characteristics are summarized in Table I. Briefly, subjects had a median age of 39.0 years (interquartile range, 18.0-59.0 years) and 61.5% were female. More than 88.0% of patients had severe persistent asthma and 25.8% had a poorly controlled disease. Furthermore, 20.4% of patients reported gastroesophageal reflux disease (GERD), 31.7% obesity, 69.3% atopy, and 27.7% recent antibiotic treatment, and 28.3% were smokers.

**FIG 1.**
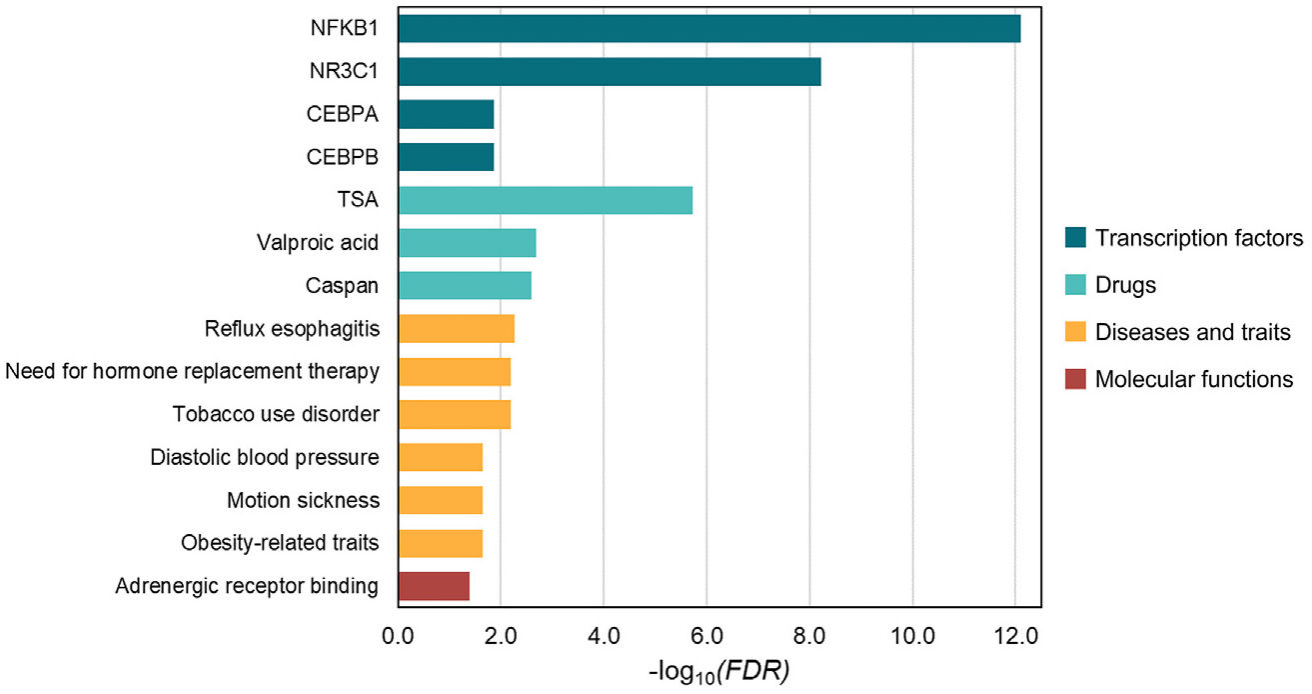
Bar plots of the most relevant significant gene-set enrichment results for each database. The −log_10_ of the *P* value adjusted by the false discovery rate (FDR) is represented on the x-axis.

Human genome-wide genotypes were imputed using the TOPMed Reference Panel, and microbiome profiling was conducted by targeted sequencing of the 16S ribosomal RNA gene (V3-V4 region), as described elsewhere.^2^ mbGWAS were conducted to test for the association of 6,296,951 genetic variants with microbiome traits through regression models adjusted for age, sex, and ancestry. We aimed to identify genes and biological pathways associated with microbiome traits (3 alpha diversity indices and 18 bacterial genera) previously associated with asthma exacerbations despite ICS treatment in the GEMAS study.^2^ Independent suggestive mbQTLs identified in a total of 24 mbGWAS (*P* < 1 × 10^−5^) were included in a gene-set enrichment analysis. After multiple comparisons adjustment (false discovery rate < 0.05), we observed an enrichment in genes previously associated with major asthma comorbidities, including reflux esophagitis (a main consequence of GERD),^10^ obesity, and smoking. Furthermore, gene-set enrichment analysis revealed that genes suggestively associated in the mbGWAS partially overlapped with genes whose expression is regulated by trichostatin A (TSA) and transcription factors, including the nuclear factor-κB (NF-κB), the glucocorticosteroid receptor or GR (encoded by the nuclear receptor subfamily 3, group C, member 1 or *NR3C1* gene), and CCAAT/enhancer-binding proteins (CEBPs) (Fig 1 and Table II). The robustness of these findings was ensured by varying the input *P*-value threshold for genetic variants selection to include in the analysis (ie, *P* < 1 × 10^−6^ and *P* < 1 × 10^−4^). Enrichment results remained significant (*P* <.05) after varying this parameter, indicating that our findings are not dependent on the arbitrary *P*-value threshold for variant selection. Stratified analyses by biological sample showed that these enrichment terms were driven by microbiome traits from different body sites (Table II).

Significant results from the salivary microbiome were followed up for replication in 158 Latino children with and without asthma from the Genes-environments & Admixture in Latino Americans (GALA II) study and 114 African Americans from the Study of African Americans, Asthma, Genes & Environments (SAGE). Their characteristics are summarized in Table I. Bioinformatic analyses were conducted using similar procedures as in the discovery phase. Enrichment in smoking, TSA, NF-κB, and GR was replicated in the mbGWAS of saliva samples from both African American and Latino children with and without asthma (4.42 × 10^−9^ ≤ *P* ≤ .008) (Table II).

GERD, obesity, and smoking are well-known risk factors for asthma susceptibility, asthma exacerbations, and corticosteroid unresponsiveness.^10-12^ Different mechanisms have been suggested to explain how GERD affects asthma, including inflammatory lung injury and vagal nerve stimulation by gastric acid.^10^ Furthermore, T_H_2 cytokines and eosinophilia might mediate the coexistence of asthma with eosinophilic esophagitis, another allergic disease that mimics GERD symptoms.^13^ However, inconsistent data have been reported about the effectiveness of GERD therapies in asthma, suggesting that the underlying mechanisms between GERD and asthma are not fully elucidated.^10^ Similarly, asthma and obesity are 2 highly heritable traits with shared mechanisms including genetic factors.^14^ The knowledge of the genetics of asthma and obesity is limited due to the polygenic character of these traits,^14^ and it has been hypothesized that genetic polymorphisms might exert an effect on the obese asthma phenotype through other omic layers.^11^ A shared feature among these 3 comorbidities is their impact on the airway, salivary, and/or gut microbiome compositions.^11,12,15^ Moreover, bacterial dysbiosis is considered a link between GERD, obesity, and asthma.^11,15^ Our study provides novel insights into the shared influence of human genetics on the upper-airway microbiome composition involved in asthma exacerbations and the development of major asthma comorbidities.

In addition, we observed an enrichment in genetic signatures related to TSA and genes regulated by NF-κB and the GR in lung inflammation bronchial epithelial cells. GR is the main mediator of the anti-inflammatory effect of glucocorticosteroids by interacting with transcriptional coactivators and corepressors (eg, histone deacetylase 2 and NF-κB).^16^ Large evidence supports that deficiencies in GR expression and activity are involved in steroid-resistant asthma.^16^ NF-κB is a proinflammatory transcription factor involved in airway inflammation in patients with asthma.^17^ Alterations in NF-κB–related pathways partially explained the heterogeneity response to asthma therapies, including ICS.^17^ TSA is an inhibitor of histone deacetylases that regulates NF-κB–driven inflammatory gene transcription and has been demonstrated to reduce airway inflammation in murine asthma models.^18^ A previous meta-GWAS in European children with asthma exacerbations despite ICS treatment showed enrichment in a TSA genetic signature.^19^ Our findings reinforce the potential therapeutic use of TSA in asthma by regulating genes involved in ICS response and microbiome composition. Nevertheless, further studies are required to evaluate the safety and efficacy of TSA as an asthma treatment. In addition, we reported enrichment in DNA motifs for CEBP-α and CEBP-β, transcription factors related to the pathophysiology of asthma.^20^ The potential corticosteroid unresponsiveness and airway cell proliferation in asthma have been related to a lack of CEBP-α in bronchial cells in these patients.^20^ CEBP members are implicated in corticosteroid response, and their expression patterns are regulated by glucocorticosteroids and bronchodilators.^20^

**FIG 2.**
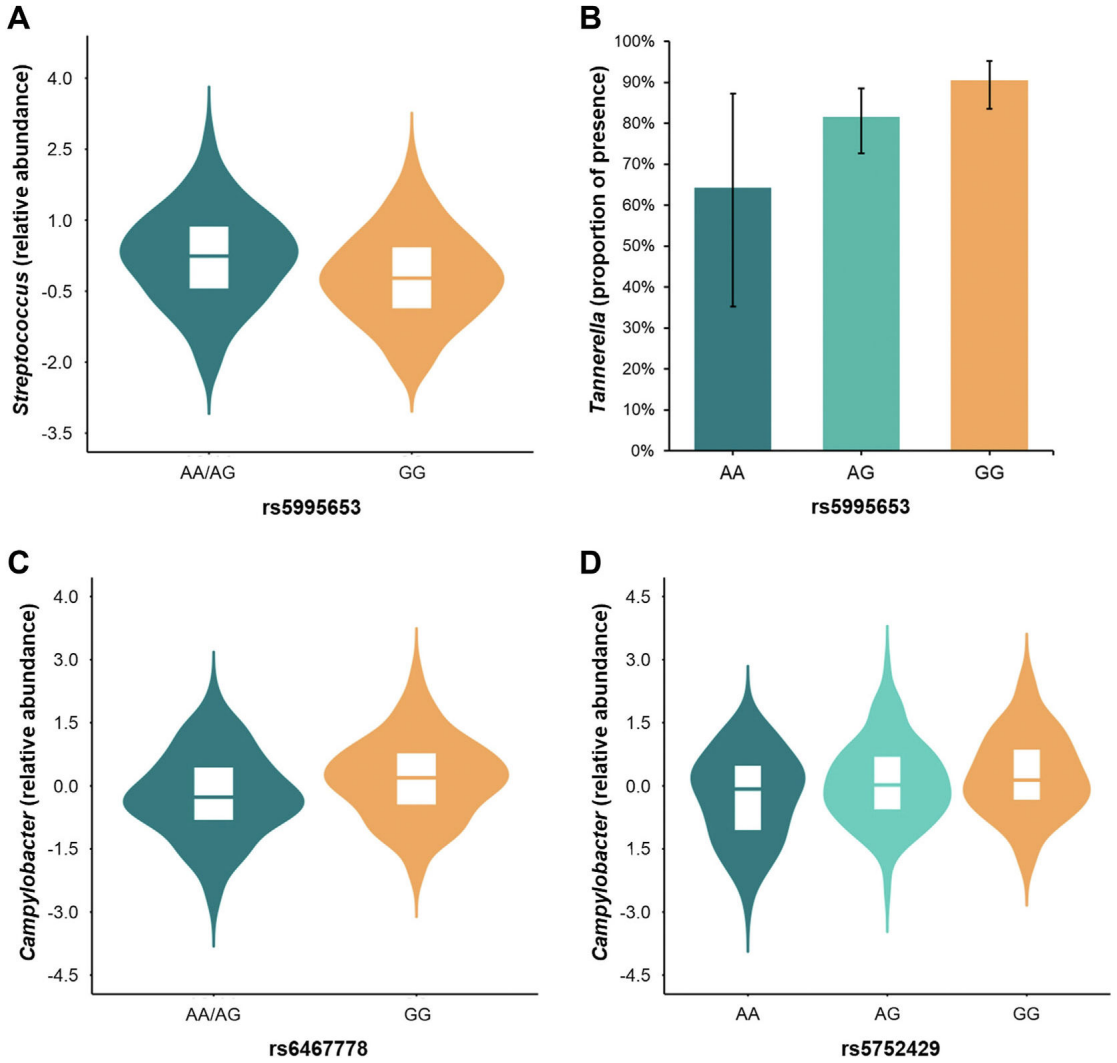
Violin plots and bar plots of the relative abundance or presence, as appropriate, of the following mbQTLs pairs: **A,** *Streptococcus*-rs5995653, **B,** *Tannerella*-rs5995653, **C,** *Campylobacter*-rs6467778, and **D,** *Campylobacter*-rs5752429. The following associations followed a dominant genetic model: *Streptococcus*-rs5995653 (*β* for the AA/AG genotype, 0.45; SE = 0.13, *P* = 5.5 × 10^−4^) and *Campylobacter*-rs6467778 (*β* for the AA/AG genotype, −0.43; SE = 0.13; *P* = 9.1 × 10^−4^).

In addition, we assessed the potential role of SNPs previously associated with ICS response by GWAS as mbQTLs of microbiome traits involved in asthma exacerbations despite ICS treatment (see Table E1 in this article’s Online Repository at www.jacionline.org). Among the 21 independent SNPs selected from the literature, 3 SNPs were identified as mbQTLs with a false discovery rate < 0.05 (Table III and Fig 2). The SNP rs5995653, located in the intergenic region of *APOBEC3B-APOBEC3C*, was associated with the relative abundance of *Streptococcus* in the nasal microbiome (*β* for the A allele, 0.34; SE, 0.11; *P* = 1.90 × 10^−3^) and the presence of *Tannerella* in the pharyngeal microbiome (*β* for the A allele, −1.06; SE, 0.33; *P* = 1.31 × 10^−3^). Moreover, the SNPs rs6467778 (*TRIM24*) (*β* for the A allele, -−0.36; SE, 0.11; *P* = 1.45 × 10^−3^) and rs5752429 (*TPST2*) (*β* for the G allele, 0.26; SE, 0.09; *P* = 4.74 × 10^−3^) were associated with the relative abundance of *Campylobacter* in the pharyngeal microbiome. All these associations remained robust in sensitivity analyses after adjusting for asthma exacerbations and potential confounders from the nasal and pharyngeal microbiome composition (all *P* < .05, Table III).

Genetic loci at *APOBEC3B-APOBEC3C*, *TRIM24*, and *TPST2* have been associated with asthma exacerbations and ICS response in multiple populations, including African Americans, Latinos, and European descendants.^21-23^ *APOBEC3B-APOBEC3C* are members of the cytidine deaminase gene family, highly expressed in the lower airways, and implicated in innate immunity and host defense against viral infections.^21^ *TRIM24* regulates IL-1 receptor (IL-1R) expression in T cells, a protein whose expression in sputum is associated with severe asthma and participates in IL-1–mediated exacerbations.^24,25^ On the other hand, *TPST2* encodes a sulfotransferase that regulates the sulfation of chemokine receptors involved in asthma T_H_2 inflammation.^23^ Our findings suggest that ICS-response–related *APOBEC3B-APOBEC3C*, *TRIM24*, and *TPST2* genetic loci affect airway bacteria associated with asthma exacerbations.

This study has several strengths. First, we integrated microbiome data from 3 different asthma-relevant body sites with human genome-wide data to conduct the first mbGWAS in patients with persistent asthma. Second, we followed reference guidelines and recommendations for microbiome profiling to ensure the robustness of sequencing assays while controlling for many potential confounders in microbiome studies.^2^ Third, the reliability and robustness of our findings were ensured by correcting for multiple comparisons, adjusting for covariates, replicating in independent populations, and conducting sensitivity and stratified analyses. Nevertheless, some limitations must be acknowledged. First, our sample size is limited to identify genome-wide significant associations. However, enrichment analyses were used as a powerful tool to identify plausible findings in the absence of genome-wide results.^8,19^ Second, although we reported evidence of replication in the salivary microbiome, we were not able to replicate the results from the nasal and pharyngeal samples because we only had access to studies with human genome-wide genotype data and 16S-ribosomal RNA–sequenced bacterial communities profiled in saliva samples. Third, the targeted metagenomic approach (16S ribosomal RNA) does not allow us to study the host genetic influence on specific bacterial species and other microorganisms involved in asthma exacerbations and ICS response.

In conclusion, genes suggestively associated with asthma exacerbation–related microbiome traits might have an influence on major asthma comorbidities development in diverse populations (ie, reflux esophagitis, obesity, and smoking). Those genetic loci are significantly more likely to be regulated by TSA and NF-κB, GR, and CEBP transcription factors than expected by chance. Finally, we reported that ICS-response–related genetic loci (*APOBEC3B-APOBEC3C*, *TR1M24*, and *TPST2*) are associated with airway bacteria related to asthma exacerbations.

## Supplementary Material

1

## Figures and Tables

**TABLE I.** Clinical and demographic characteristics of study populations

Variable	N	GEMAS (n = 257)	N	SAGE (n = 114)*	N	GALA II (n = 158)*
Age (y)	257	39.0 (18.0-59.0)	114	14.8 (12.3-18.5)	158	12.2 (9.7-14.3)
Sex (female)	257	158 (61.5)	114	64 (56.1)	158	88 (55.7)
Pre-FEV_1_ (%)	242	85.5 (70.6-101.7)	57	94.7 (88.6-102.1)	39	99.8 (82.9-116.6)
Pre-FVC (%)	241	89.1 (74.6-103.6)	57	100.1 (93.1-113.3)	39	102.8 (87.5-115.9)
FEV_1_/FVC (%)	241	79.4 (72.3-84.7)	57	82.8 (77.9-86.2)	39	86.7 (82.6-90.9)
Asthma	257	257 (100)	114	57 (50.0)	158	39 (24.7)
Asthma control	236		57		39	
Well controlled		118 (50.0)		11 (19.2)		13 (33.3)
Partially controlled		57 (24.2)		23 (40.4)		14 (35.9)
Poorly controlled		61 (25.8)		23 (40.4)		12 (30.8)
Asthma severity	251		57		39	
Mild asthma		12 (4.8)		42 (73.7)		27 (71.1)
Moderate asthma		18 (7.2)		15 (26.3)		11 (28.9)
Severe asthma		221 (88.0)		0 (0)		0 (0)
Atopy	257	178 (69.3)	46	27 (58.7)	0	NA
BMI category	243		57		39	
Normal weight		81 (33.3)		3 (5.3)		0 (0)
Overweight		85 (35.0)		4 (7.0)		0 (0)
Obesity		77 (31.7)		50 (87.7)		39 (100)
GERD	255	52 (20.4)	0	NA	0	NA
Smoking exposure†	244	69 (28.3)	57	13 (22.8)	0	NA
ICS treatment in the past year	257	250 (97.3)	57	21 (36.8)	39	9 (23.1)
Antibiotic use	257	71 (27.7)	0	NA	0	NA
Season of sampling	257		0		0	
Spring		13 (5.1)		NA		NA
Summer		50 (19.5)		NA		NA
Autumn		46 (17.9)		NA		NA
Winter		148 (57.6)		NA		NA
Cavities	256	52 (20.3)	0	NA	0	NA
Smoke‡	253	4 (1.6)	0	NA	0	NA
Liquid intake‡	249	13 (5.2)	0	NA	0	NA

Variables were recorded during patient recruitment. Descriptive statistics are represented by the median (interquartile range) for continuous variables and the count (proportion) for categorical variables.

*BMI,* Body mass index; *FVC*, forced vital capacity; *GEMAS*, Genomics and Metagenomics of Asthma Severity; *NA*, not available; *SAGE*, Study of African Americans, Asthma, Genes & Environments.

* Asthma-related variables are available only for asthma cases in the analyzed subsets from SAGE and GALA II studies.

† Smoker or secondhand smoker.

‡ Variables recording activities in the previous 30 min to biological sample collection.

**TABLE II.** Summary results of the main significant GSEA results

	Discovery									Replication			
	All samples (GEMAS)			Saliva		Pharyngeal		Nasal		SAGE		GALA II	
Term	OR	*P*	FDR	OR	*P*	OR	*P*	OR	*P*	OR	*P*	OR	*P*
**Traits: PheWeb 2019**													
Reflux esophagitis	36.3	**1.11** × **10**^−**5**^	**0.005**	46.1	**.001**	NA	NA	37.7	**.002**	NA	NA	NA	NA
Need for HRT	24.2	**4.38** × **10**^−**5**^	**0.006**	32.1	**.002**	21.3	**.049**	12.4	.082	17.5	**.007**	NA	NA
Tobacco use disorder	23.1	**5.14** × **10**^−**5**^	**0.006**	14.5	**.070**	43.7	**.001**	11.9	.085	16.7	**.008**	26.8	**.003**
**Traits: GWAS Catalog 2019**													
Diastolic blood pressure	12.3	**9.21** × **10**^−**5**^	**0.022**	6.5	.148	29.9	**2.01** × **10**^−**4**^	5.3	.177	3.5	.251	5.6	.167
Motion sickness	18.1	**1.19** × **10**^−**4**^	**0.022**	24.6	**.004**	NA	NA	20.1	**.005**	NA	NA	10.2	.098
Obesity-related traits	2.9	**2.51** × **10**^−**4**^	**0.022**	2.9	**.024**	1.3	.482	3.7	**.002**	1.8	.112	2.0	.114
**Drugs: Drug signatures database**													
TSA	2.9	**1.17** × **10**^−**9**^	**1.87** × **10**^−**6**^	3.0	**1.38** × **10**^−**4**^	2.8	**.003**	3.0	**2.01** × **10**^−**5**^	2.1	**.001**	2.4	**.001**
Valproic acid	2.1	**3.92** × **10**^−**6**^	**0.002**	2.0	**.006**	1.6	.107	2.6	**9.71** × **10**^−**5**^	1.5	**.028**	1.8	**.012**
Caspan	3.5	**7.39** × **10**^−**6**^	**0.003**	2.9	**.025**	2.6	.077	4.1	**3.81** × **10**^−**4**^	1.2	0.387	2.5	**.044**
**Gene ontologies: GO Molecular Function 2021**													
AR binding	27.1	**3.15** × **10**^−**4**^	**0.040**	49.2	**.001**	32.0	**.033**	NA	NA	NA	NA	NA	NA
**TFs: Genome Browser PWM**													
CEBPA	5.2	**4.63** × **10**^−**5**^	**0.013**	2.8	.164	4.0	.095	9.0	**2.78** × **10**^−**5**^	1.5	.377	NA	NA
CEBPB	5.0	**6.56** × **10**^−**5**^	**0.013**	2.7	.175	8.2	**.002**	5.9	**.002**	3.0	**.048**	1.2	.582
**TFs: ChIP-seq Enrichment Analysis (ChEA 2022)** *													
NFKB1	3.8	**1.24** × **10**^−**15**^	**7.80** × **10**^−**13**^	3.7	**3.07** × **10**^−**6**^	3.5	**1.66** × **10**^−**4**^	4.0	**3.92** × **10**^−**8**^	3.4	**4.42** × **10**^−**9**^	2.5	**4.52** × **10**^−**4**^
NR3C1	3.2	**7.57** × **10**^−**11**^	**6.02** × **10**^−**9**^	3.6	**1.31** × **10**^−**5**^	1.6	.153	3.9	**2.84** × **10**^−**7**^	2.6	**3.17** × **10**^−**5**^	4.0	**2.56** × **10**^−**7**^

Only the top 3 significant results for each database in the discovery phase are summarized (a complete list is described in the Online Repository).

Significant *P* values are highlighted in boldface.

*AR*, Adrenergic receptor; *ChIP*, chromatin immunoprecipitation; *FDR*, false discovery rate; *GEMAS*, Genomics and Metagenomics of Asthma Severity; *GSEA*, gene-set enrichment analysis; *HRT*, hormone replacement therapy; *PWM*, Position Weight Matrix; *SAGE*, Study of African Americans, Asthma, Genes & Environments; *NA*, not available; *OR*, odds ratio; *TF*, transcription factor.

* ChIP-seq assays in human bronchial epithelial cells from human lung inflammation models are reported in this table.

**TABLE III.** Summary statistics and sensitivity analyses of SNPs previously associated with ICS response identified as mbQTLs

						Main model†			Adjusted by exacerbations‡		Adjusted by microbiome confounders§	
Genus	rsID	Position*	Gene	A1/A2	MAF	*β* (SE)	*P* value	FDR	*β* (SE)	*P* value	*β* (SE)	*P* value
**Nasal microbiome**												
*Streptococcus*	rs5995653	22:39008244	*APOBEC3B-APOBEC3C*	A/G	0.27	0.34 (0.11)	.002	0.040	0.34 (0.11)	.002	0.33 (0.12)	.005
**Pharyngeal microbiome**												
*Tannerella*	rs5995653	22:39008244	*APOBEC3B-APOBEC3C*	A/G	0.28	−1.06 (0.33)	.001	0.027	−1.07 (0.33)	.001	−1.04 (0.35)	.003
*Campylobacter*	rs6467778	7:138493477	*TRIM24*	A/G	0.26	−0.36 (0.11)	.001	0.031	−0.33 (0.11)	.003	−0.35 (0.12)	.003
*Campylobacter*	rs5752429	22:26833014	*TPST2*	G/A	0.42	0.26 (0.09)	.005	0.050	0.25 (0.09)	.006	−0.24 (0.09)	.010

*A1*, Effect allele; *A2*, noneffect allele; *β*, regression coefficient; *FDR*, false discovery rate; *MAF*, minor allele frequency; *rsID*, reference SNP cluster ID.

* Position based on GRCh38/hg38 build.

† The main model was adjusted for age, sex, and ancestry.

‡ Main model including as covariate asthma exacerbations in the past 6 months despite ICS treatment.

§ Main model including the following covariates: antibiotic use, sequencing pool, sampling season, body mass index, and smoke in the past 30 min for nasal samples; and antibiotic treatment, cavities, and liquid intake in the past 30 min for pharyngeal samples.

## Data Availability

The data that support the findings and conclusions of this study are reported and available in the main text of this article and the Zenodo repository (https://doi.org/10.5281/zenodo.7472532). The data reported in the Zenodo repository will be openly available after the publication of the article. This article includes a Methods section in the article’s Online Repository at www.jacionline.org. Demultiplexed sequencing reads of the 16S ribosomal RNA gene used in this study are publicly available and can be downloaded from the Sequence Read Archive database under the accession number PRJNA878647.

## Key messages

- Genes associated with exacerbation-related microbiome traits are related to the susceptibility to major asthma comorbidities (ie, reflux esophagitis, obesity, and smoking).
- TSA and multiple transcription factors have a potential therapeutic interest in asthma exacerbations treatment by targeting genes that regulate exacerbation-related airway bacteria.
- ICS-response-related genetic loci (*APOBEC3B-APOBEC3C*, *TRIM24*, and *TPST2*) affect airway bacteria associated with asthma exacerbations.

## Abbreviations used

CEBP: CCAAT/enhancer-binding protein
GEMAS: Genomics and Metagenomics of Asthma Severity
GALA II: Genes-environments & Admixture in Latino Americans
GERD: Gastroesophageal reflux disease
ICS: Inhaled corticosteroid
mbGWAS: Microbiome genome-wide association study
mbQTL: Microbiome quantitative trait locus
NR3C1: Nuclear receptor subfamily 3, group C, member 1
NF-κB: Nuclear factor-κB
PC: Principal component
SNP: Single nucleotide polymorphism
TSA: Trichostatin A
