# Supplementary material for: Human genetics influences microbiome composition involved in asthma exacerbations despite inhaled corticosteroid treatment
Source: J Allergy Clin Immunol. Author manuscript; Available in PMC 2023 Sep 26. (PMC10522330; doi:10.1016/j.jaci.2023.05.021)
Supplement: 1 [file NIHMS1931148-supplement-1.pdf]

METHODS

Study population

In this study, we included individuals with available microbiome and genotype data enrolled in the GEMAS study (identifier NCT04501926 in the clinicalTrials.gov database of the National Institutes of Health). A full description of the rationale and design of the study, as well as the characteristics of recruited patients, has been previously reported.[E1] Briefly, GEMAS is a case-control study aimed to identify genetic variants and changes in the human microbiome associated with asthma exacerbations. European-descent males and females aged between 8 and 80 years with a physician diagnosis of asthma based on the Global INitiative for Asthma 2020 guidelines[E2] were recruited in allergy and respiratory medicine hospital units in the Canary Islands and the Basque Country (Spain). This study was approved by the correspondent ethics committees (approvals 29/17 and PI2019077), and all patients and parents, when necessary, gave their written consent or assent as appropriate.

Human genome-wide genotyping

Blood samples were collected from all recruited patients in EDTA tubes for human DNA extraction. Genomic DNA was isolated using the Illustra blood genomicPrep Mini Spin Kit (GE Healthcare, Amersham, UK) following the manufacturer's instructions. DNA samples were randomized in 96-well plates according to case and control status (asthma exacerbations) and genotyped using the Infinium Global Screening Array-24 kit v3.0 (Illumina, San Diego, Calif) by Centro Nacional de Genotipado (CEGEN-ISCIII). A total of 654,027 SNPs across the whole genome were genotyped. Raw intensity data obtained from the microarray were preprocessed using the GenomeStudio 2.0 software (Illumina). Clustering and variant calling were conducted using the Infinium Global Screening Array v3.0 manifest. The PLINK Input Report Plug-in v2.1.4 was used to export only non-zeroed SNP genotype data into PLINK (PED/MAP) files format (n = 650,181).

Quality control (QC) of genotype data was conducted following standard guidelines using PLINK 1.9 and R, as described elsewhere.[E3-E6] Previous to the standard QC, genotype data were preprocessed by filtering out potential problematic SNPs (variants without chromosome annotation, indels, duplicates, multiallelic variants, and monomorphic SNPs in our population). The Infinium Global Screening Array v3.0 manifest (Illumina) and the SNPNexus database were used for SNP annotation.[E7]

A QC of genetic markers was carried out by removing SNPs with a genotyping call rate < 95%. Regarding the QC of individuals, first, no discordance between the reported sex and the biological sex estimated from genotype data was identified for any samples. Second, no sample with a high missing genotype rate (>5%) was identified. Third, on the basis of heterozygosity rate in autosomal chromosomes, we did not identify any evidence of potential cross-sample contamination (upper heterozygosity rate outliers) or the presence of inbreeding (lower heterozygosity rate outliers). Fourth, first- and second-degree–related individuals (Pihat ≥ 0.2) were identified and excluded (n = 5). Finally, a principal component (PC) analysis of genotype data did not identify any individuals with large differences in ancestry. The optimal number of genotype PCs required to correct for ancestry in subsequent analyses was previously addressed by including 1-by-1 each PC in stepwise GWAS of asthma exacerbations using genotype data.[E4] Based on quantile-quantile plots, 2 PCs were selected as appropriate to correct for population stratification in the GEMAS study. A total of 282 individuals remained after the QC (257 with microbiome data). Genotype data were annotated into GRCh38/hg38 genome assembly and imputed against the TOPMed reference panel to increase the available number of genetic markers.[E8] SNPs in Hardy-Weinberg disequilibrium were filtered out (n = 5,035). This resulted in 6,296,951 common SNPs with minor allele frequency greater than or equal to 5% and a good imputation quality score ($r^2 > 0.3$).

Bacterial microbiome profiling

Saliva, pharyngeal, and nasal samples were collected from patients of the GEMAS study. A full description of biological sample collection and storage, DNA extraction, sequencing libraries preparation, and sequencing reads processing has been previously described elsewhere.[E9] Briefly, 1 mL of saliva was collected using the Oragene OMNIgene ORAL OM501 tubes (DNA Genotek, Inc, Ottawa, Ontario, Canada), while pharyngeal and nasal samples were obtained using sterile swabs stored in AMIES transport medium (DeltaSwab Amies, Deltalab, Barcelona, Spain). Samples were collected during regular medical check-ups and stored at −20°C until they were processed. Genomic DNA was extracted using the Pathogen Lysis Tubes S kit (Qiagen, Hilden, Germany) for mechanical prelysis of bacterial cells, and the QIAamp UCP Pathogen Mini kit (Qiagen) for DNA isolation and purification. Negative controls for each sample type, including molecular biology–grade water instead of a biological sample, were processed under the same conditions to control for potential environmental and reagent contamination. All samples were processed by the same researcher in a laminar flow cabin under controlled cleanliness conditions (use of sterilized expendable material and daily disinfection of equipment and surfaces with ethanol 70% and 20-minute ultraviolet irradiance).

Paired-end reads of the V3-V4 region of the 16S ribosomal RNA (16S rRNA) gene were obtained using the MiSeq sequencing platform (Illumina) from 789 biological samples. The QIIME2 bioinformatic platform[E10] was used for denoising sequencing reads and amplicon sequence variants (ASVs) clustering using the DADA2 pipeline,[E11] taxonomy assignment and depletion of nonbacterial sequences using the SILVA rRNA reference database (v138),[E12] sequence alignment, and phylogenetic tree construction.[E13,E14] The negative controls of DNA extraction, as well as negative controls of PCR (unprocessed molecular biology–grade water), were sequenced to control for potential environmental contamination. The 13 sequenced negative controls had an average of 76 ± 46 denoised reads. The *decontam* R package was used to identify contaminant ASVs on the basis of their prevalence in negative controls and biological samples,[E15] but only 5 contaminant ASVs were identified in the 3 types of biological samples. Moreover, the estimation of alpha diversity indices after rarefying sequencing reads and relative abundance estimation of bacterial genera were conducted in R using the *phyloseq* R package.[E5,E16] Only samples with more than 10,000 denoised reads were retained for subsequent analyses. We ensured the robustness and reproducibility of microbiome profiling by including DNA from a simulated mock community with known microbial composition as a positive control

(ZymoBIOMICS Microbial Community DNA Standard I, ZymoResearch, Irvine, Calif), and replicates of biological samples for DNA extraction (n = 10) and library preparation (n = 4). We observed a high correlation between the observed and expected bacterial composition of the positive controls included in each sequencing pool, and both DNA extraction and PCR replicates of biological samples were highly correlated among them. A full assessment of negative and positive control samples, as well as replicates of biological samples, is reported elsewhere.[E9]

Microbiome GWAS

The contribution of the human genetic variation to the microbiome composition was inspected through mbGWAS. We aimed to identify genes and biological pathways associated with microbiome traits (3 alpha diversity indices and 18 bacterial genera) previously associated with asthma exacerbations despite ICS treatment in the GEMAS study.[E9] These include the richness (observed ASVs), Shannon's index, and Faith's phylogenetic index in saliva and nasal samples, and the relative abundance of *Absconditabacteriales*, *Bifidobacterium*, and *Capnocytophaga* in saliva samples, *Selenomonas*, *Atopobium*, *Tannerella,* and *Campylobacter* in pharyngeal samples, and *Porphyromonas*, *Prevotella*, *Streptococcus*, *Fusobacterium*, *Lawsonella*, *Dialister*, *Neisseriaceae spp*., *Neisseria*, *Leptotrichia*, *Actinomyces*, and *Rothia* in nasal samples. Thus, a total of 24 mbGWAS were conducted to identify mbQTLs of these alpha diversity indices and bacterial taxa. The relative abundance of bacterial genera and alpha diversity metrics were normalized using the inverse normal transformation. Briefly, this method ranks order and transforms the data in quantiles to then reestimate the true values as Z scores that follow a normal distribution. Because zero inflation could hamper variable normalization and linear modeling, those genera with an elevated number of zeroes across samples (>5%) were dichotomized into presence or absence categories.[E17] The genera that were dichotomized included *Absconditabacteriales*, *Actinomyces*, *Bifidobacterium*, *Dialister*, *Fusobacterium*, *Lawsonella*, *Leptotrichia*, *Neisseria*, *Neisseriaceae spp*., *Porphyromonas*, *Prevotella*, *Rothia*, and *Tannerella*. We tested for the association between microbiome traits and SNP allele dosages in PLINK 2.0 through a linear or logistic regression model, as appropriate, adjusted by age, sex, and ancestry (the first 2 PCs of genotype data).[E6] mbGWAS results were filtered by removing variants with extreme values for regression coefficients and/or SEs (absolute values ≥ 10).

Enrichment analyses

Gene-set enrichment analyses (GSEA) were conducted to identify potential overrepresentations of genes involved in human diseases and traits, drug genetic signatures, and/or biological processes. We included in the GSEA the genes where independent mbQTLs were annotated from the salivary, nasal, and pharyngeal mbGWAS using a threshold of $P < 1 \times 10^{-5}$. Independent signals were identified using the clumping method (clumps of 500 kb; $r^2 > 0.2$) in PLINK 1.9.[E6] The most significant SNP for each clump was retained and annotated to the nearest gene using GREAT v4.0.[E18] After removing duplicates, the resulting genes were collapsed into 1 dataset combining the mbGWAS from the 3 biological samples. GSEA was performed using the Enrichr tool, and only significant results were reported after multiple comparison corrections (false discovery rate < 0.05).[E19] To provide stable and robust findings, GSEA was reassessed by varying the P-value thresholds for selection of SNPs (ie, $P < 1 \times 10^{-6}$ and $P < 1 \times 10^{-4}$), and only the main findings that remained significant using these 2 alternative thresholds were reported ($P < .05$). To evaluate the contribution of each biological sample, the main results were further assessed in stratified enrichment analysis for the type of biological sample (ie, saliva, pharyngeal, and nasal samples).

We used the databases PheWeb 2019 and GWAS Catalog 2019 to inspect enrichment in traits and human diseases, the Drug signatures database for drug genetic signatures (DSigDB), Genome Browser Position Weight Matrix (PWM), and ChIP Enrichment Analysis (ChEA 2022) databases for transcription factors binding sites, and Gene Ontology (GO) Molecular Function 2021 for biological processes.

Assessment of SNPs involved in ICS response as mbQTLs

Using the data generated in the mbGWAS, we attempted to identify whether SNPs previously associated with ICS response by GWAS were mbQTLs of the microbiome traits we reported to be associated with asthma exacerbations despite ICS use.[E9] A literature search was conducted using the PubMed database to identify GWAS of ICS response conducted in patients with asthma until December 2022 (n = 15).[E20-E34] We selected the SNPs declared to be associated with ICS response for each study and filtered out those in high linkage disequilibrium and with a minor allele frequency < 0.05 in our population. A total of 21 independent SNPs associated with ICS response were included in the analyses (Table E1). A false discovery rate < 0.05 was used to correct for multiple testing.

Significant results were plotted using violin plots (for relative abundance–based mbGWAS) and bar plots (for presence/absence-based mbGWAS). On the basis of graphical plots and regression models, we investigated the genetic model (ie, additive, dominant, or recessive) that best fitted our data based on the Akaike information criterion. To ensure the robustness of our associations, we performed sensitivity analyses adjusting by (1) asthma exacerbations despite ICS treatment and (2) all potential confounders of microbial communities previously identified in the GEMAS study.[E9] These potential confounders included age, antibiotics usage, cavities, and liquid intake in the past 30 minutes before sample collection for pharyngeal samples, and age, sex, sequencing pool, body mass index, season, and smoke in the past 30 minutes before sample collection for nasal samples.

Replication in saliva samples

We sought to replicate our findings from the salivary microbiome in 2 parallel case-control studies of childhood and youth asthma with available human genome-wide genotype data and amplicon-sequenced bacterial communities profiled in saliva samples.[E35] These included Latino and African American children with and without asthma from the Genes-environments & Admixture in Latino Americans (GALA II) study and the Study of African American, Asthma, Genes & Environments (SAGE),[E35] respectively. Full information regarding the study design and enrollment of patients is reported elsewhere.[E36,E37] Briefly, patients were recruited between 2006 and 2014 through

clinical and community-based recruitment centers in the San Francisco Bay Area (SAGE) and different areas of the United States and Puerto Rico (GALA II). Participants were aged between 8 and 21 years and self-identified as African Americans (SAGE) or Hispanics/Latinos (GALA II). All their 4 grandparents were also self-identified as the same population. Asthma cases were defined by a doctor's diagnosis of asthma, recent use of asthma medication, or occurrence of 2 or more asthma symptoms (cough, wheeze, or shortness of breath) in the 2 years before enrollment. SAGE and GALA II were approved by the Human Research Protection Program Institutional Review Board (IRB) at the University of California, San Francisco (UCSF) (UCSF-IRB No. 10-02877 and No. 10-00889, respectively). All parents and participants provided signed written consent and assent as appropriate.

All details about microbiome profiling have been previously described.[E35] Briefly, saliva samples were collected using the Oragene DNA Discover OGR-500 self-collection kits (DNA Genotek, Inc, Stittsville, Ontario, Canada) to ensure the stability of microbial communities. Bacterial communities were profiled by targeted sequencing of the V4 16S rRNA region, including also negative and positive controls of sequencing libraries. In this study, Illumina-sequenced reads were processed using the same bioinformatic protocol described in the GEMAS study to minimize the heterogeneity among the studies. Genotyping of human variation was carried out with the Axiom LAT1 array (817,810 genetic variants) and the Axiom LAT1 Array Plus HLA (812,715 genetic variants) (Affymetrix, Santa Clara, Calif) and the QC procedure was similar to the one described for the GEMAS study.[E38] Imputation was also carried out using the TOPMed reference panel.[E8] mbGWAS, estimation of independent SNPs, gene annotation, and GSEA were also conducted following the same pipeline as described for the GEMAS study. In this case, regression models were also adjusted for asthma status. Population stratification was corrected using 2 and 3 PCs in SAGE and GALA II, respectively. The *Bifidobacterium* genus was absent in 95% of Latino individuals, so this genus was excluded from the analyses in GALA II. Replication was declared only for those terms that showed a significant enrichment association ($P < .05$) in both replication populations.

REFERENCES

E1. Perez-Garcia J, Hernández-Pérez JM, González-Pérez R, Sardón O, Martin-Gonzalez E, Espuela-Ortiz A, et al. The Genomics and Metagenomics of Asthma Severity (GEMAS) study: rationale and design. J Pers Med 2020;10:123.
E2. Global Initiative for Asthma. Global strategy for asthma management and prevention. Fontana (WI): Global Initiative for Asthma; 2020.
E3. Anderson CA, Pettersson FH, Clarke GM, Cardon LR, Morris AP, Zondervan KT. Data quality control in genetic case-control association studies. Nat Protoc 2010;5:1564-73.
E4. Herrera-Luis E, Ortega VE, Ampleford EJ, Sio YY, Granell R, de Roos E, et al. Multi-ancestry genome-wide association study of asthma exacerbations. Pediatr Allergy Immunol 2022;33:e13802.
E5. R Core Team. R: a language and environment for statistical computing. Vienna, Austria: R Foundation for Statistical Computing; 2020.
E6. Chang CC, Chow CC, Tellier LC, Vattikuti S, Purcell SM, Lee JJ. Second-generation PLINK: rising to the challenge of larger and richer datasets. Gigascience 2015;4:7.
E7. Oscanoa J, Sivapalan L, Gadaleta E, Dayem Ullah AZ, Lemoine NR, Chelala C. SNPnexus. a web server for functional annotation of human genome sequence variation (2020 update). Nucleic Acids Res 2020;48:W185-92.
E8. Taliun D, Harris DN, Kessler MD, Carlson J, Szpiech ZA, Torres R, et al. Sequencing of 53,831 diverse genomes from the NHLBI TOPMed Program. Nature 2021;590:290-9.
E9. Perez-Garcia J, González-Carracedo M, Espuela-Ortiz A, Hernández-Pérez JM, González-Pérez R, Sardón-Prado O, et al. The upper-airway microbiome as a biomarker of asthma exacerbations despite inhaled corticosteroid treatment. J Allergy Clin Immunol 2023;151:706-15.
E10. Bolyen E, Rideout JR, Dillon MR, Bokulich NA, Abnet CC, Al-Ghalith GA, et al. Reproducible, interactive, scalable and extensible microbiome data science using QIIME 2. Nat Biotechnol 2019;37:852-7.
E11. Callahan BJ, McMurdie PJ, Rosen MJ, Han AW, Johnson AJA, Holmes SP. DADA2: high-resolution sample inference from Illumina amplicon data. Nat Methods 2016;13:581-3.
E12. Quast C, Pruesse E, Yilmaz P, Gerken J, Schweer T, Yarza P, et al. The SILVA ribosomal RNA gene database project: improved data processing and web-based tools. Nucleic Acids Res 2013;41:D590-6.
E13. Price MN, Dehal PS, Arkin AP. FastTree 2 – approximately maximum-likelihood trees for large alignments. PLoS One 2010;5:e9490.
E14. Katoh K, Misawa K, Kuma KI, Miyata T. MAFFT: a novel method for rapid multiple sequence alignment based on fast Fourier transform. Nucleic Acids Res 2002;30:3059-66.
E15. Davis NM, Proctor DiM, Holmes SP, Relman DA, Callahan BJ. Simple statistical identification and removal of contaminant sequences in marker-gene and metagenomics data. Microbiome 2018;6:226.
E16. McMurdie PJ, Holmes S. phyloseq: an R package for reproducible interactive analysis and graphics of microbiome census data. PLoS One 2013;8:e61217.
E17. Hughes DA, Bacigalupe R, Wang J, Rühlemann MC, Tito RY, Falony G, et al. Genome-wide associations of human gut microbiome variation and implications for causal inference analyses. Nat Microbiol 2020;5:1079-87.
E18. McLean CY, Bristor D, Hiller M, Clarke SL, Schaar BT, Lowe CB, et al. GREAT improves functional interpretation of cis-regulatory regions. Nat Biotechnol 2010;28:495-501.
E19. Kuleshov MV, Jones MR, Rouillard AD, Fernandez NF, Duan Q, Wang Z, et al. Enrichr: a comprehensive gene set enrichment analysis web server 2016 update. Nucleic Acids Res 2016;44:W90-7.
E20. Park TJ, Park JS, Cheong HS, Park BL, Kim LH, Heo JS, et al. Genome-wide association study identifies ALLC polymorphisms correlated with FEV_1 change by corticosteroid. Clin Chim Acta 2014;436:20-6.
E21. Hernandez-Pacheco N, Gorenjak M, Jurgec S, Corrales A, Jorgensen A, Karimi L, et al. Combined analysis of transcriptomic and genetic data for the identification of loci involved in glucocorticosteroid response in asthma. Allergy 2021;76:1238-43.
E22. Hernandez-Pacheco N, Vijverberg SJ, Herrera-Luis E, Li J, Sio YY, Granell R, et al. Genome-wide association study of asthma exacerbations despite inhaled corticosteroid use. Eur Respir J 2021;57:2003388.
E23. Hernandez-Pacheco N, Gorenjak M, Li J, Repnik K, Vijverberg SJ, Berce V, et al. Identification of ROBO2 as a potential locus associated with inhaled corticosteroid response in childhood asthma. J Pers Med 2021;11:733.
E24. Park HW, Dahlin A, Tse S, Duan QL, Schuemann B, Martinez FD, et al. Genetic predictors associated with improvement of asthma symptoms in response to inhaled corticosteroids. J Allergy Clin Immunol 2014;133:664-9.e5.
E25. Dahlin A, Denny J, Roden DM, Brilliant MH, Ingram C, Kitchner TE, et al. CMTR1 is associated with increased asthma exacerbations in patients taking inhaled corticosteroids. Immunity Inflamm Dis 2015;3:350-9.
E26. Wang Y, Tong C, Wang Z, Wang Z, Mauger D, Tantisira KG, et al. Pharmacodynamic genome-wide association study identifies new responsive loci for glucocorticoid intervention in asthma. Pharmacogenomics J 2015;15:422-9.
E27. Tantisira KG, Damask A, Szefler SJ, Schuemann B, Markezich A, Su J, et al. Genome-wide association identifies the T gene as a novel asthma pharmacogenetic locus. Am J Respir Crit Care Med 2012;185:1286-91.
E28. Tantisira KG, Lasky-Su J, Harada M, Murphy A, Litonjua AA, Himes BE, et al. Genomewide association between GLCCI1 and response to glucocorticoid therapy in asthma. N Engl J Med 2011;365:1173-83.
E29. Kan M, Diwadkar AR, Shuai H, Joo J, Wang AL, Ong MS, et al. Multiomics analysis identifies BIRC3 as a novel glucocorticoid response-associated gene. J Allergy Clin Immunol 2022;149:1981-91.
E30. Levin AM, Gui H, Hernandez-Pacheco N, Yang M, Xiao S, Yang JJ, et al. Integrative approach identifies corticosteroid response variant in diverse populations with asthma. J Allergy Clin Immunol 2019;143:1791-802.
E31. Wu AC, Himes BE, Lasky-Su J, Litonjua A, Peters SP, Lima J, et al. Inhaled corticosteroid treatment modulates ZNF432 gene variant's effect on bronchodilator response in asthmatics. J Allergy Clin Immunol 2014;133:723-8.e3.
E32. Ortega VE, Daya M, Szefler SJ, Bleecker ER, Chinchilli VM, Phipatanakul W, et al. Pharmacogenetic studies of long-acting beta agonist and inhaled corticosteroid responsiveness in randomised controlled trials of individuals of African descent with asthma. Lancet Child Adolesc Heal 2021;5:862-72.

E33. Hernandez-Pacheco N, Farzan N, Francis B, Karimi L, Repnik K, Vijverberg SJ, et al. Genome-wide association study of inhaled corticosteroid response in admixed children with asthma. Clin Exp Allergy 2019;49:789-98.
E34. Wang AL, Lahousse L, Dahlin A, Edris A, McGeachie M, Lutz SM, et al. Novel genetic variants associated with inhaled corticosteroid treatment response in older adults with asthma. Thorax 2023;78:432-41.
E35. Espuela-Ortiz A, Lorenzo-Diaz F, Baez-Ortega A, Eng C, Hernandez-Pacheco N, Oh SS, et al. Bacterial salivary microbiome associates with asthma among African American children and young adults. Pediatr Pulmonol 2019;54:1948-56.
E36. Borrell LN, Nguyen EA, Roth LA, Oh SS, Tcheurekdjian H, Sen S, et al. Childhood obesity and asthma control in the GALA II and SAGE II studies. Am J Respir Crit Care Med 2013;187:697.
E37. Nishimura KK, Galanter JM, Roth LA, Oh SS, Thakur N, Nguyen EA, et al. Early-life air pollution and asthma risk in minority children. The GALA II and SAGE II studies. Am J Respir Crit Care Med 2013;188:309-18.
E38. Espuela-Ortiz A, Herrera-Luis E, Lorenzo-Díaz F, Hu D, Eng C, Villar J, et al. Role of sex on the genetic susceptibility to childhood asthma in Latinos and African Americans. J Pers Med 2021;11:1140.

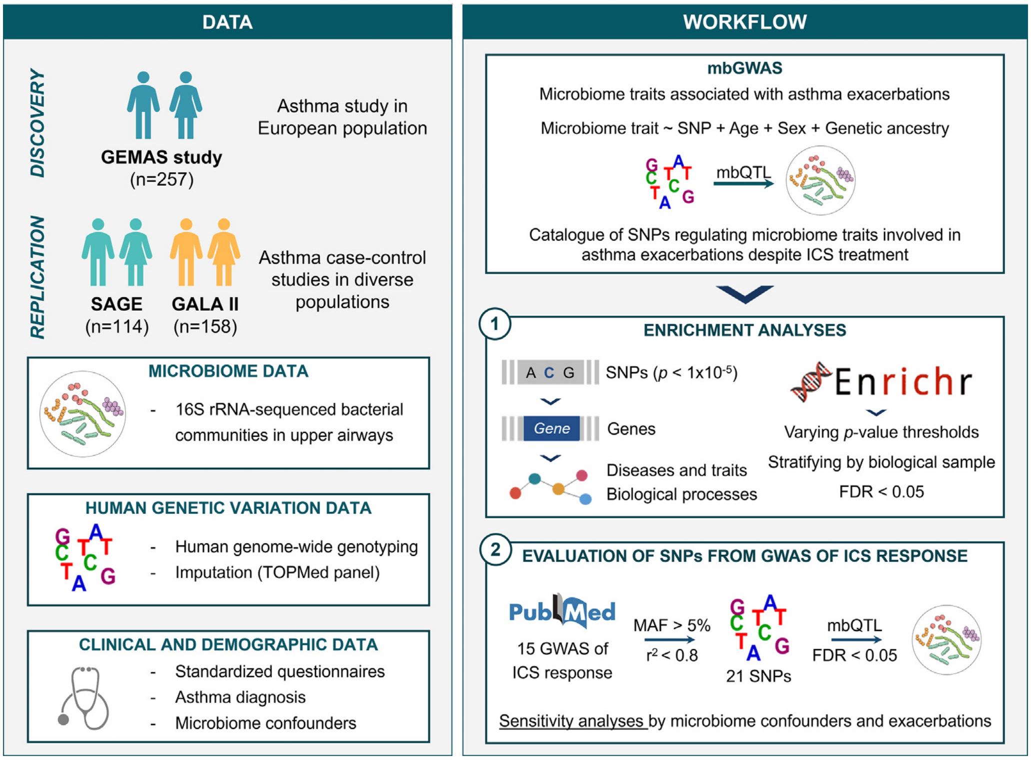

FIG E1. Schematic overview of the available data and workflow of this study. The salivary, pharyngeal, and nasal microbiome data were combined with genome-wide human genetic variation, focusing on microbiome traits previously associated with asthma exacerbations in the GEMAS study. We generated a catalog of mbQTLs through mbGWAS. Enrichment analyses were used to identify whether genetic signatures involved in microbiome shaping are related to other phenotypes, biological processes, or drug mechanisms of action. Furthermore, we evaluated whether SNPs previously associated with ICS response in the literature by GWAS were mbQTLs of the upper-airway microbiome.

TABLE E1. SNPs previously associated with ICS response by GWAS included in the mbQTL analysis

rsID	Chr	Position*	Gene	Reference
rs11123610	2	3675436	*ALLC*	Park et al[E20]
rs11681246	2	33241553	*LTBP1*	Hernandez-Pacheco et al[E21]
rs67026078	3	55128671	*CACNA2D3-WNT5A*	Hernandez-Pacheco et al[E22]
rs1166980	3	76879839	*ROBO2*	Hernandez-Pacheco et al[E23]
rs10044254	5	15783487	*FBXL7*	Park et al[E24]
rs2395672	6	37460801	*CMTR1*	Dahlin et al[E25]
rs6924808	6	97910699	*MMS22L-FBXL4*	Wang et al[E26]
rs6456042	6	166121254	*PDE10A-T*	Tantisira et al[E27]
rs37972	7	7967878	*UMAD1-GLCCI1*	Tantisira et al[E28]
rs2691529	7	78173958	*MAGI2*	Dahlin et al[E25]
rs6467778	7	138493477	*TRIM24*	Dahlin et al[E25]
rs4271056	9	38232046	*SHB-ALDH1B1*	Dahlin et al[E25]
rs1353649	11	20232053	*NAV2-HTATIP2*	Wang et al[E26]
rs2846858	11	102306442	*BIRC3*	Kan et al[E29]
rs3827907	14	20770639	*EDDM3B*	Levin et al[E30]
rs9303988	18	6667584	*L3MBTL4-ARHGAP28*	Dahlin et al[E25]
rs9955411	18	24494756	*HRH4-ZNF521*	Tantisira et al[E27]
rs3752120	19	52048768	*ZNF432-ZNF841*	Wu et al[E31]
rs279728	20	46451782	*ELMO2-ZNF334*	Dahlin et al[E25]
rs5752429	22	26833014	*TPST2*	Ortega et al[E32]
rs5995653	22	39008244	*APOBEC3B-APOBEC3C*	Hernandez-Pacheco et al[E33]

Only independent SNPs reported in each study were included. Note that 3 independent SNPs (rs138717703, rs73399224, and rs138717703) had an MAF <0.05 in the GEMAS study and were excluded.

MAF, Minor allele frequency; *rsID*, Reference SNP cluster ID.

*Position based on GRCh38/hg38 build.
